# New species and a new genus of Philopotamidae from the Andes of Bolivia and Ecuador (Insecta, Trichoptera)

**DOI:** 10.3897/zookeys.780.26977

**Published:** 2018-08-08

**Authors:** Ralph W. Holzenthal, Roger J. Blahnik, Blanca Ríos-Touma

**Affiliations:** 1 Department of Entomology, University of Minnesota, 1980 Folwell Avenue, 219 Hodson Hall, St. Paul, Minnesota 55108 USA University of Minnesota St. Paul United States of America; 2 Facultad de Ingenierías y Ciencias Aplicadas, Ingeniería Ambiental, Grupo de Investigación en Biodiversidad Medio Ambiente y Salud – BIOMAS – Universidad de Las Américas, Campus Queri, Quito, Ecuador Universidad de Las Américas Quito Ecuador; 3 Instituto Nacional de Biodiversidad, Quito, Pichincha, Ecuador Instituto Nacional de Biodiversidad Quito Ecuador

**Keywords:** Andes, Bolivia, Choco, Ecuador, South America

## Abstract

A new genus and species of Philopotamidae (Philopotaminae), *Aymaradellaboliviana*, is described from the Bolivian Andes of South America. The new genus differs from other Philopotaminae by the loss of 2A vein in the hind wing and, in the male genitalia, the synscleritous tergum and sternum of segment VIII, and the elongate sclerotized dorsal processes of segment VIII. The first record of *Hydrobiosella* (Philopotaminae) in the New World is also provided with a new species from the Andes of Ecuador, *Hydrobiosellaandina*. In addition, a new species of the Neotropical genus *Chimarrhodella* (Chimarrinae), *Chimarrhodellachoco*, is described from the Choco-Andean region of Ecuador, and *Chimarrhodellaperuviana* (Ross) is recorded from Ecuador for the first time. Lastly, *Wormaldiaimbrialis* (Philopotaminae), new species, is described, also from the Ecuadorian Choco.

## Introduction

The cosmopolitan family Philopotamidae, with approximately 1300 species, is especially species rich in the tropics, where many new species have been described recently (e.g., Blahnik et al 2009, Johanson and Oláh 2012, Dumas and Nessimian 2013). Currently, there are 22 genera in three subfamilies: Chimarrinae (three genera), Philopotaminae (18 genera), and Rossodinae (one genus) (Blahnik 2005, Holzenthal et al. 2007, Morse 2017) (Table 1). The subfamily Philopotaminae was the subject of a revision by Ross (1956). He treated many of what are now recognized as genera, as subgenera of a nearly cosmopolitan genus, *Sortosa*, subsequently emended to *Dolophilodes* by Ulmer (1957) due to name priority. *Dolophilodes*, as formulated by Ross, was based mainly on plesiomorphic characters. The subgenera were largely restricted and endemic to different continental areas: South America, South Africa, Australia, Asia, and North America. Subsequently, some of these were raised to generic status (e.g., Neboiss 1977) and the process was completed by Blahnik (2005), who raised all of the remaining subgenera to genera, based on the criteria initially used by Ross to establish them. However, a great many species have been described in the interim since Ross’s revision, both to those originally placed in *Dolophilodes*, or to other genera recognized by Ross or described subsequently (Blahnik 2005, Gibon 2014). The characters used by Ross no longer easily diagnose some genera. This is especially true of the genera *Sortosa* and *Hydrobiosella*, in which the generic assignment of new species has been mostly based on their geographic location. Clearly, what is needed is a revision of the entire subfamily. Pending this, generic assignments are at best provisional for some taxa, made in accordance with existing taxonomic criteria. Ideally, however, their placement should facilitate an eventual generic and subfamily reassessment.

**Table 1.** Genera of Philopotamidae, distribution, and approximate number of species. Available synonyms in italics. *=new continental record). Adapted from Holzenthal et al. (2007), Morse (2017).

**Chimarrinae** Rambur, 1842	***Kisaura*** Ross, 1956
***Chimarra*** Stephens, 1829	Oriental
Cosmopolitan	ca. 50 species
ca. 750 species	***Neobiosella*** Wise, 1958
***Chimarrhodella*** Lestage, 1925	Australasian (New Zealand)
Neotropical	1 species
13 species	***Philopotamus*** Stephens, 1829
***Edidiehlia*** Malicky, 1993	Palearctic (Europe)
Oriental (Sumatra)	10 species
1 species	***Ranarijaodes*** Gibon, 2014
**Philopotaminae** Stephens, 1829	Afrotropical (Madagascar)
***Alterosa*** Blahnik, 2005	3 species
Neotropical (southeast Brazil)	***Sisko*** Ross, 1956
39 species	Nearctic
***Aymaradella* gen. n.**	2 species
Neotropical (Bolivia)	***Sortosa*** Navas, 1918
1 species	Neotropical (Patagonia)
***Cryptobiosella*** Henderson, 1983	20 species
Australasian (New Zealand)	***Thylakion*** Barnard, 1934
4 species	Afrotropical
***Dolophilodes*** Ulmer, 1909	4 species
*Hisaura* Kobayashi, 1980	***Wormaldia*** McLachlan, 1865
*Trentonius* Betten & Mosely, 1940	*Cabreraia* Enderlein, 1929
Nearctic, Palearctic, Oriental	*Doloclanes* Banks, 1937
ca. 60 species	*Dolophiliella* Banks, 1930
***Dolomyia*** Schmid, 1991	*Dolophilus* McLachlan, 1868
Oriental (India)	*Gatlinia* Ross, 1948
1 species	*Nanagapetus* Tsuda, 1942
***Dolopsyche*** Schmid, 1991	*Paragapetus* Banks, 1914
Oriental (India)	Cosmopolitan
1 species	ca. 200 species
***Fumonta*** Ross, 1956	***Xenobiosella*** Henderson, 1983
Nearctic (eastern USA)	Australasian (New Zealand)
1 species	1 species
***Gunungiella*** Ulmer, 1913	**Rossodinae** Oezdikmen & Darilmaz, 2008
Oriental	***Rossodes*** Oezdikmen & Darilmaz, 2008
ca. 100 species	Afrotropical (Madagascar)
***Hydrobiosella*** Tillyard, 1924	16 species
*Zelobiosella* Mosely, 1953	
Australasian, Neotropical* (Ecuador)	
ca. 30 species	

Neotropical areas, especially the Tropical Andes, harbor an incredible and unexplored Trichoptera diversity (Ríos-Touma et al. 2017). More than 3000 species of Trichoptera have been recorded from the region, including 377 extant species in five genera of Philopotamidae: *Alterosa* (39 species), *Chimarra* (256 species), *Chimarrhodella* (12 species), *Sortosa* (20 species), and *Wormaldia* (50 species) (Holzenthal and Calor 2017). Here, we describe a new genus of Philopotaminae, *Aymaradella*, from Bolivia and we provide the first continental record for *Hydrobiosella*, previously known only from the Australasian region (Holzenthal et al. 2007; Cartwright 2010), with a new species from the Andes of Ecuador. Also, we describe a new species of *Chimarrhodella* from the Choco-Andean region of Ecuador and provide new country records of *Chimarrhodellaperuviana* (Ross). Finally, *Wormaldiaimbrialis*, new species, a member of a group of species related to *W.prolixa* Flint, is also described from the Choco of Ecuador. Larvae have not been described for any of the Neotropical species in the genera treated in this paper, except *Wormaldia*.

## Materials and methods

Adult specimens of *Aymaradellaboliviana*, new genus, new species, *Chimarrhodellachoco*, new species, and *Wormaldiaimbrialis*, new species, were collected at UV fluorescent lights placed adjacent to streams. Lights were hung in front of a white bed sheet and powered from a small 12 volt, sealed, lead-acid battery. Additional specimens of *A.boliviana* and of *Hydrobiosellaandina*, new species, were borrowed from the National Museum of Natural History, Smithsonian Institution; no information on habitat or collecting method is available. Males and females were associated indirectly by common occurrence and overall similarity in body size and color.

Adult specimens were prepared and examined following standard methods for pinned and alcohol preserved material (Blahnik and Holzenthal 2004; Blahnik et al. 2007). Length of forewing was measured from base to apex, and is presented as the range followed by the number of specimens measured. For specimens collected by us, male genitalia were soaked in 85% lactic acid heated to 125 °C for 20 min to dissolve internal soft tissues. An Olympus BX41 compound microscope outfitted with a drawing tube was used to examine specimens and to aid the rendering of detailed pencil drawings of genitalic structures. Pencil sketches were scanned and placed in Adobe Illustrator (Creative Cloud version) to serve as a template for vector illustrations. Morphological terminology follows that of Blahnik (2005) for genitalia and Holzenthal et al. (2007) for wing venation. Each specimen was affixed with a barcode label (4-mil polyester, 8 × 14 mm, code 49) bearing a unique alphanumeric sequence beginning with the prefix UMSP to serve as a specimen identifier for upload of collection and specimen data to the University of Minnesota Insect Collection (UMSP) database.

Types of the new species and other material examined are deposited in the University of Minnesota Insect Collection, St. Paul, Minnesota, USA (**UMSP**), the Museo Ecuatoriano de Ciencias Naturales, Insituto Nacional de Biodiversidad, Quito, Ecuador (**MECN**), the Museo de Historia Natural "Noel Kempff Mercado", Santa Cruz de la Sierra, Bolivia (**UASC**), and the National Museum of Natural History, Smithsonian Institution, Washington, DC (**NMNH**).

## Systematics

### Aymaradella gen. n.

Taxon classificationAnimaliaTrichopteraPhilopotamidae

http://zoobank.org/57368721-91ED-44F0-BA96-151D7A2C4335

Figs 1 , 2 , 3

#### Type species.

*Aymaradellaboliviana*, new species

#### Diagnosis.

*Aymaradella*, new genus, can be distinguished from any other genus of Philopotaminae by the loss of 2A vein in the hind wing, the synscleritous tergum and sternum of segment VIII, and the elongate sclerotized dorsal processes of segment VIII.

This species has the general venational attribute of lacking the second anal vein in the hind wing, a character used to define the genera *Wormaldia* and *Gunungiella*. However, it is very distinctly different from either of those genera in overall form, and completely unlike any described species of *Wormaldia* from either North or South America. In particular, the completely fused segment VIII, with elongate dorsal processes, is unique among species in Philopotaminae.

#### Description.

Adult. Head relatively short and rounded, postparietal sclerite short (ca. ½ diameter of eye). Spur formula 2:4:4. Maxillary palps 5-segmented, segment II with apicomesal bristles, labial palps 3-segmented. Venation complete for Philopotamidae (forewings with forks I-V, hind wing lacking fork IV); forewing with discoidal cell relatively short, forks I and II approximately sessile, crossveins *s*, *r-m*, and *m* hyaline and nearly linear, 3A looped to 2A, 2A to 1A, intersecting in proximal half of vein. Fork I of hind wing with short stem, fork II sessile, 1A and 3A intersecting wing margin, 2A missing.

Male. Sternum VII with short, rounded mesoventral process (rather than flattened, spatulate process typical of *Wormaldia*). Segment VIII synscleritous, expanded anterodorsally, dorsal margin with pair of elongate processes. Segment IX synscleritous, narrowing dorsally. Tergum X simple in structure and entire, wide basally, narrowing apicaly, with numerous sensilla and/or short setae. Preanal appendages elongate, narrow, digitiform, emerging near base of tergum X. Inferior appendages bi-segmented, linear, apical segment with mesal pad of short stiff setae. Phallic apparatus simple in structure, without spines or inclusions.

Female. Genitalia elongate and tapering from segment VII; segment VII longer than preceding segment, with small ventral process at midlength; segment VIII nearly as long as segment VII, tapering, not synscleritous, sternite with lateral pair of very elongate, narrow apodemes. Segment IX very short, anterolateral margin with pair of elongate, narrow apodemes, extending anterad. Segment X composed of pair of elongate, narrow sensillate lobes, each with short apical cercus. Vaginal apparatus membranous, without evident sclerites.

#### Etymology.

The name Aymara is considered feminine and refers to the people and language of Bolivia; the suffix is considered to be a diminutive and makes the name euphonious with *Chimarrhodella* and *Hydrobiosella*, other philopotamids previously and newly known from the Neotropics.

### Aymaradella boliviana sp. n.

Taxon classificationAnimaliaTrichopteraPhilopotamidae

http://zoobank.org/48C88BD8-662F-47B9-8585-BA1BE0DB2F79

Figs 1 , 2 , 3

#### Diagnosis.

Diagnosed by the characteristics of the genus as discussed above.

#### Description.

Adult. Forewing length (male) 5.5–5.8 mm (n = 2); (female) 5.8–6.3 mm (n = 2). Spur formula 2:4:4. Overall color, including wings and antennae, light brown, legs yellowish brown. Head short and rounded, eyes with short setae between facets, postparietal sclerite short (ca. ½ diameter of eye). Palps short; maxillary palp with segment I very short, segments II and IV short, II with apicomesal bristles, III only moderately elongate, V longer than III. Forewing with forks I-V, hind wing with forks I-III and V (IV absent). Forewing with discoidal cell relatively short, forks I and II approximately sessile, crossveins *s*, *r-m*, and *m* hyaline and nearly linear, 3A looped to 2A, 2A to 1A, intersecting in proximal half of vein. Fork I of hind wing with short stem, fork II sessile, 1A and 3A intersecting wing margin, 2A missing.

Male. Abdomen with segments through VII with sternites generally setose, tergites V-VII with setae confined to (more or less) linear row on posterior margin, each seta with more or less evident, desclerotized area at base. Sternum VII with short rounded mesoventral process from posterior margin, directed posterad and positioned posterior to sclerotized line that extends near the posterior margin from the mesoventral process to midlateral margin of sternite. Segment VIII synscleritous, ventrally ca. ½ length of sternite VII, widening anterodorsally to width subequal to tergum VII; as viewed dorsally, with anterior margin concavely invaginated, mesally with pair of elongate, narrow, sclerotized processes, with apices acutely narrowed and somewhat laterally projecting, extending from near anterior margin of segment beyond posterior margin of segment IX; dorsomesal part of segment, from lateral margin of posteromesal invagination to posterior of segment, only weakly sclerotized or submembranous. Segment IX synscleritous, ventral margin subequal in length to sternum VIII, evenly narrowed from posterior margin to narrow, sclerotized, invaginated, dorsomesal strap; posterior of segment weakly sclerotized or submembranous. Tergum X simple in structure, elongate, narrow, slightly widened near base and uniformly narrowed apically; apex rounded, basally with pair of small rounded protuberances, each with 2-3 short stiff setae; dorsal surface with short setae or seta-like sensilla, declining in size apically, extreme apex with cluster of small sensilla. Preanal appendages elongate, narrow, emerging near base of tergum X; as viewed dorsally, somewhat mesally curved, emerging near base of tergum X. Inferior appendages elongate and relatively narrow, widest near base of basal segment; apex of apical segment slightly widened, with cluster of short, stiff setae on apicomesal surface. Phallic apparatus with phallobase more or less tubular, with usual basodorsal projection, relatively short, simple, tapering from base to apex; phallotremal sclerite small, indistinct, endotheca simple, without associated spines or ornamentation.

Female. Genitalia very elongate, tapering from segment VII; segment VII much longer than preceding segment (ca. 1½ x length), ventral margin with very small, acute, mesoventral process at midlength; segment VIII nearly as long as segment VII, tapering, not synscleritous, sternite with lateral pair of very elongate, narrow apodemes, extending from anterdorsal margin, apodemes nearly 1½ x length of segment VII. Segment IX very short, (apparently comprised of tergum only), anterolateral margin with pair of very elongate, narrow apodemes, extending anterad, length ca. 1½ x length of segment VIII; posterior margin with pair of elongate, narrow sensillate lobes (segment X), each with short apical cercus. Vaginal apparatus membranous, only indistinctly evident.

**Figure 1–2.**
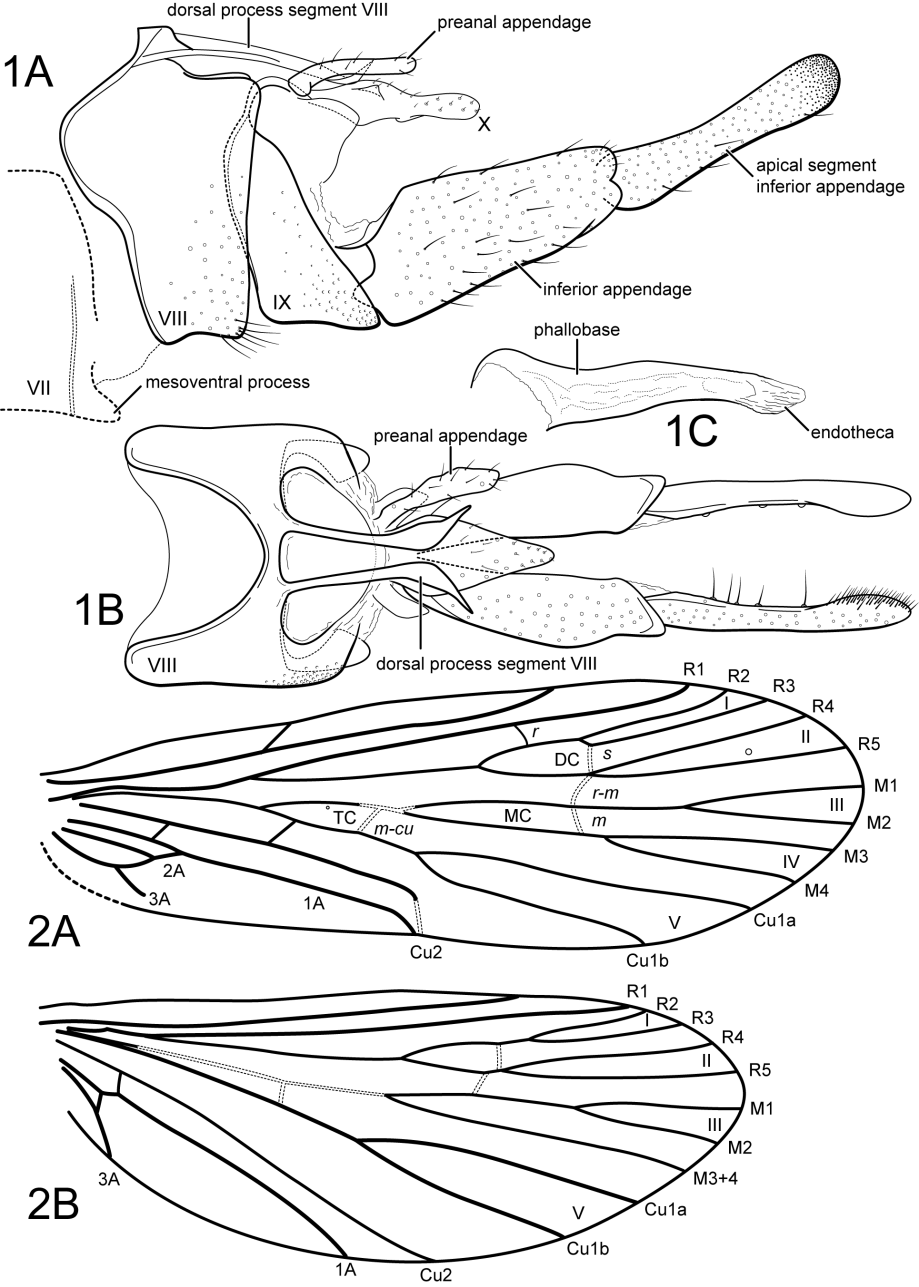
**1***Aymaradellaboliviana* gen. n., sp. n. Male genitalia **A** segments VII-X, lateral **B** segments VIII-X, dorsal **C** phallus, lateral. **2***Aymaradellaboliviana*, gen. n., sp. n. Male wings **A** forewing **B** hind wing.

**Figure 3.**
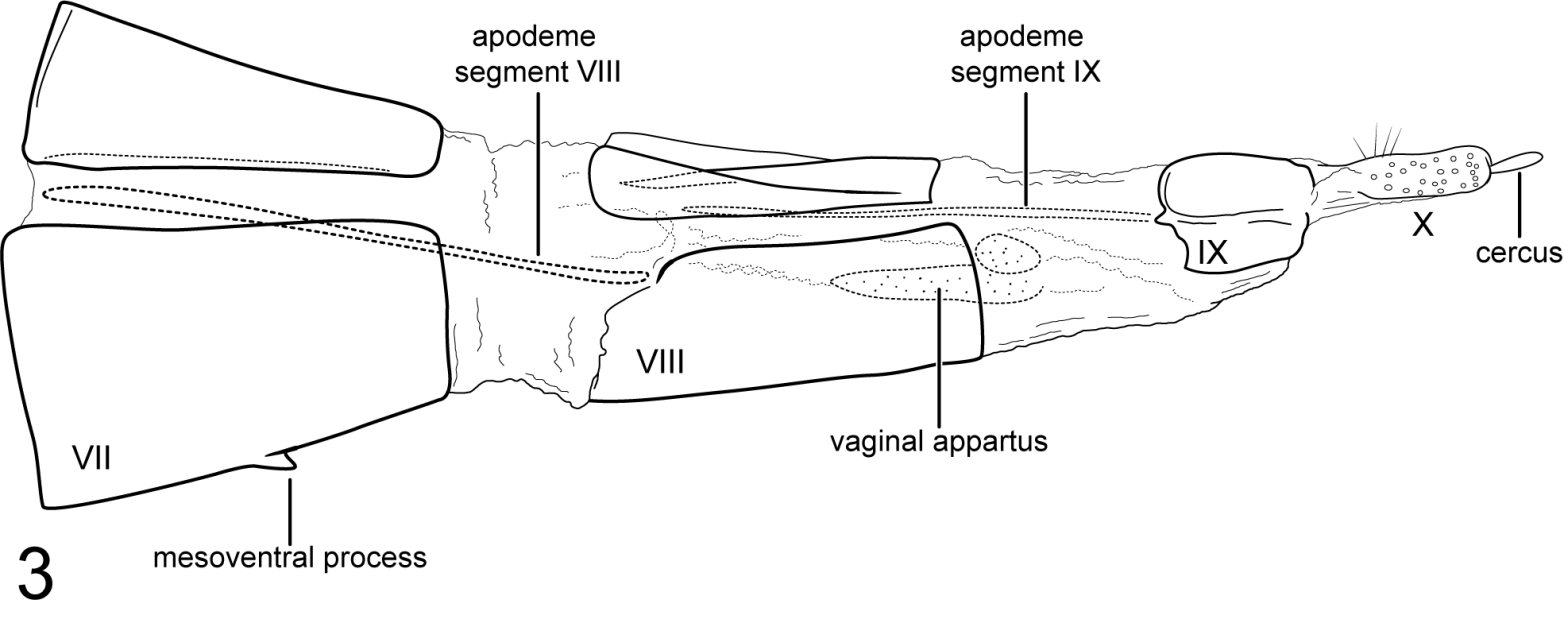
*Aymaradellaboliviana*, gen. n., sp. n. Female genitalia, lateral.

#### Holotype.

**Male. BOLIVIA: La Paz**: quebrada del Río Zongo, 1400 m, 24–30.x.1984, L.E. Peña G. (UMSP000136162) (NMNH). **Paratypes**: same data as holotype, 1 male, 2 females (NMNH); **BOLIVIA: La Paz**: PN-ANMI [Parque Nacional y Área Natural de Manejo Integrado] Cotapata, Estacíon Biológica Tunquini, Quebrada El Padrini, 16°12.193’S, 67°50.692’W, el. 1343 m, 06-07.xii.2004, Robertson, Valdivia, 9 males, 2 females (UMSP, UASC).

#### Etymology.

Named for the country where the species was discovered.

#### Remarks.

The generic placement of this new species from Bolivia, which is unlike other Neotropical species of Philopotaminae, requires a discussion of the world fauna, particularly of the genus *Wormaldia*.

*Wormaldia*, in general, has a cosmopolitan distribution, excluding the Australian region. The Central and South American species of the genus agree in general form with other species in the genus (Muñoz-Quesada and Holzenthal 2015). *Wormaldia* was treated comprehensively by Ross (1956), who recognized two subgenera, *Wormaldia* and *Doloclanes* and a single isolated species from the Philippines, *W.recta* Ulmer. Ross also treated the genus *Gunungiella*, which is characterized by apomorphic and often bizarre modifications of the inferior appendages and a reduced venation, namely with forks III and IV of the forewing absent and also with distinctive reductions and modifications of the hind wing. Only a very few species of *Gunungiella* were known at the time Ross did his revision, but this has changed, especially with a revision of the genus by Schmid (1968), who added 34 new species, mostly from India. Many additional species have been added since then, from India and Southeast Asia to Indonesia (e.g., Huisman 1993, Melnitsky and Ivanov 2010). Ross considered the possibility that *W.recta* might be related to *Gunungiella*, primarily because of its loss of fork IV in the forewing. However, Schmid questioned this placement and decided that the unusual species was probably correctly allied with *Wormaldia*, and thus independently lost fork IV in the forewing. Schmid agreed with Ross that it was a unique and isolated species with many primitive attributes.

The generic name *Doloclanes* has been variably treated by different authors. The genus was established by Banks (1937) for two species from the Philippines. It was reduced to a subgenus of *Wormaldia* by Ross (1956), who provided a rather informal diagnosis and recognized nine species in the subgenus, four of them new. He also reduced the generic names of *Nanagapetus* Tsuda and *Gatlinia* Ross to synonyms. The latter were based on the species *Nanagapetuskisoensis* Tsuda and *Gatliniamohri* Ross, monotypic genera from Japan and Eastern North America, respectively. *Doloclanes* was subsequently raised to full generic status by Schmid (1991), who provided a more formal diagnosis and described eleven new species from India. The genus was subsequently reduced to synonymy with *Wormaldia* by Sun and Malicky (2002), based on the variability they observed concerning Ross’s venational characters in some species (e.g., *W.quadriphylla*). They stated that even a subgeneric status was doubtful. However, no mention was made of Schmid’s work and the more extensive list of diagnostic characters he provided. At present, *Doloclanes* is considered a synonym of *Wormaldia*, but its status should probably be considered provisional until a more formal assessment is made. The single North American species placed in *Doloclanes*, *W.mohri*, was subsequently treated by Muñoz-Quesada and Holzenthal (2008), who considered it a member of the *thryia* species group of *Wormaldia*. However, the species rather clearly demonstrates all of the diagnostic criteria used by Schmid to recognize species of *Doloclanes*. Since none of these characters apply to the new species from Bolivia treated here, it cannot be considered a member of the genus *Doloclanes*, regardless of its formal status.

Placement of the new species from Bolivia also requires a consideration of the species of *Wormaldia* from Africa. Ross (1956) placed two African species in what he called the *kyana* group (in the subgenusWormaldia), commenting that they had a peculiar morphology and that they were probably an isolated lineage close to the ancestor of *Wormaldia*. Additional species from Madagascar and the African mainland have been discovered since then, most with elongate and filamentous preanal appendages. Gibon (2014), in describing a number of new species from Madagascar, subsequently divided the African species into the *kyana* group, including four mainland African species, and the *pauliani* group, including eleven species from Madagascar. The Madagascar species seem to consistently lack fork IV in the forewing (as in *W.recta* from the Philippines), as indicated in illustrations of new species from Madagascar by Johanson (2010). However, at least some mainland species have complete and primitive venation for Philopotamidae.

Given the unusual morphology of *W.recta* and the African species of *Wormaldia*, their inclusion in the genus should possibly be reconsidered, especially since the genus is otherwise morphologically uniform and well characterized. It should be noted also that the genus *Thylakion*, from South Africa, while not having the 2A vein of the forewing obsolete, does have it reduced to a stub, much as in the unrelated genus *Chimarrhodella*. *Thylakion* also has processes lateral to tergum X. Its possible relationship to African species of *Wormaldia* should probably be considered. However, these are questions independent of the placement of our new species from Bolivia. Given the fact that it possesses none of the apomorphic characters of the unusual species of *Wormaldia* from either the Philippines or Africa, and has an unusual set of characters of its own, we believe that the most reasonable way to treat the taxon is to assign it to a new genus. The designation also points out the need to include the species in subsequent studies of relationships among and within genera of Philopotaminae, which is sorely needed.

### Chimarrhodella choco sp. n.

Taxon classificationAnimaliaTrichopteraPhilopotamidae

http://zoobank.org/294225D2-880D-497D-944C-5082317FFFE4

Figs 4 , 5

#### Diagnosis.

This new species is distinctive. Especially diagnostic is the coloration, with yellowish head and thorax and brown wings; also diagnostic are the relatively short curved inferior appendages and the characteristic shape of tergum X. The general structure of the inferior appendages, with acute apices, is like that of *C.costaricensis* and species of the *peruviana* group. The linear row of bristle-like setae on the inferior appendage found in the new species, generally characterizes species in both the *galeata* and *peruviana* groups, but apparently not *C.ornata* Blahnik. However, like *C.ornata*, *C.choco* has short preanal appendages and a pale testaceous or yellowish head and thorax. Also it has a relatively simple phallic structure, without the highly pleated endotheca and pleated dorsal expansion that characterizes other species. The short female genitalia resemble those of species in the *galeata* group.

#### Description.

Adult. Forewing length (male) 5.7–6.7 mm (n = 9); (female) 6.1–7.0 (n = 4). Spur formula 2:4:4. Head, thorax, legs, and palps yellowish, setae of head and thorax golden (yellowish orange), postparietal sclerite and mesal suture of head slightly infuscated; basal antennal segments and basal half of subsequent segments brownish, apices yellowish; abdomen grayish brown; setae of wings, tibial spurs (and scant setae of legs) light to medium brown. Maxillary palps relatively short, segment I very short, globular, segments II and IV relatively short, subequal, segment II with apicomesal bristles, segments III and V subequal, moderate in length. Head very elongate (as in *C.galeata* Blahnik & Holzenthal, 1992, fig 1A), distinctly flattened, posparietal sclerite elongate (ca. as long as diameter of eye). Wings held flattened over dorsum of body, nearly horizontal. Wing venation typical for genus, forewing fork IV absent; hind wing 2A reduced to short stub (Blahnik and Holzenthal 1992).

Male. Abdominal segments through VII with relatively sparse, fine setae on sterna and terga, denser posteriorly. Segment VIII relatively short, ca. ½ length of preceding segments, sternum with granulate surface sculpture (posterior margin with fringe of setae, plus premarginal setae mesally); setation of tergum like preceding segments. Segment IX relatively simple in structure, longer than segment VIII, anterior margin nearly linear (slightly concave), ventral margin weakly produced; posterior margin sinuously invaginated at level of inferior appendages, dorsal part setose, broadly rounded and somewhat produced; as viewed dorsally, with dorsal margin narrow, concavely narrowed from both anterior and posterior margins. Tergum X relatively short, bilobed, separated by short, narrow, submembranous mesal lobe; apices of lobes capitate, each with short, acute lateral projection; base of each lobe with prominent lateral conical sensillum; apices of lobes with numerous small sensilla. Preanal appendages short, rounded, fused with posterodorsal margin of tergum IX. Inferior appendages moderately elongate, more or less linear, but distinctly curved dorsad, tapering from base to apex, apex with short, acute projection, lateral margins densely setose, mesal margin with linear row of short, stiff setae, extending nearly length of appendage. Phallobase relatively short, with basodorsal expansion, ventral apex projecting, weakly sclerotized, broadly rounded as viewed dorsally or ventrally, internally with 2 short conical sclerotized spines, bases often slightly enlarged; endotheca emerging from tubular structure, distinctly sclerotized basally, membranous and down-curved apically, with short, tubular, weakly sclerotized phallotremal sclerite apically; dorsally with additional hood-like membranous lobe, simple in structure and not at all pleated.

Female. Segment VIII relatively short, with short, but distinct apodemes on anterolateral margin, posterior margin with 3 pairs of setal warts, posteroventral margin with mesal, setose, strap-like projection, fused basally to sternum IX. Tergum IX relatively short, with pair of elongate, narrow apodemes from anterior margin (shorter than length of segment VIII), posterior margin with ca. 3 elongate setae, clustered laterally on either side of segment IX, apically with pair of bulbous, setose projections (tergum X), each with short apical cercus and short setose projection from basoventral margin. Sternum IX short, rounded, lightly sclerotized, not extending beyond ventral strap of segment VIII. Vaginal apparatus membranous, indistinct, apically with small cup-like sclerite.

**Figure 4–5.**
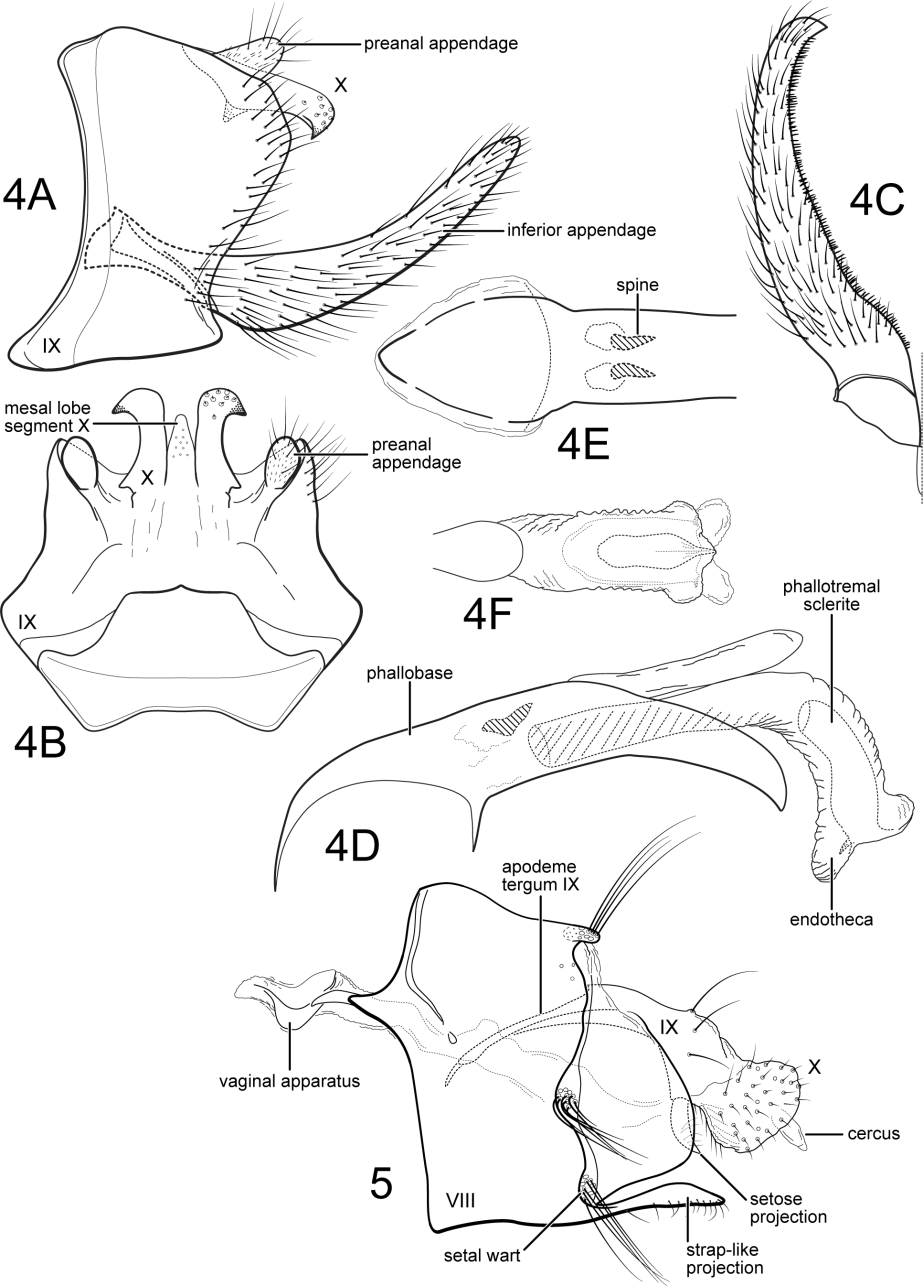
**4***Chimarrhodellachoco* sp. n. Male genitalia **A** segments IX–X, lateral **B** segments IX–X, dorsal **C** inferior appendages, ventral **D** phallus, lateral **E** base of phallus, dorsal **F** apex of phallus, dorsal **5***Chimarrhodellachoco*, sp. n. Female genitalia, lateral.

#### Holotype.

**Male. ECUADOR: Imbabura**: Reserva Los Cedros, tributary to Rio Los Cedros, 00.30374°N, 78.78229°W, 1312 m, 18-19.x.2011, Holzenthal, Ríos, Encalada, Acosta [UMSP000098416] (UMSP). **Paratypes: ECUADOR: Imbabura**: Reserva Los Cedros, small stream near station, 00.31127°N, 78.78150°W, 1460 m, 17.x.2011, Holzenthal, Ríos, Encalada, Acosta, 5 males (UMSP, MECN); Reserva Los Cedros; Rio de la Plata, 00.32495°N, 78.78084°W, 1587 m, 15.iii.2012, Ríos-Touma, Bragado, Policha, 1 male (UMSP); Reserva Los Cedros, Rio Los Cedros, 00.30359°N, 78.78233°W, 1312 m, 18-19.x.2011, Holzenthal, Ríos, Encalada, Acosta, 1 female (UMSP); **Pichincha**: Amagusa Reserve (private), Río Mashpi Chico “alto”, 00.15487°N, 78.85316°W, 1180 m, 17.i.2015, Holzenthal, Huisman, Ríos-Touma, 2 males, 2 females (NMNH, MECN); Río Malimpia in Mashpi Lodge, 00.17063°N, 78.88804°W, 700 m, 18.i.2015, Holzenthal, Huisman, Ríos-Touma, 1 female (UMSP).

#### Etymology.

Named for the Choco-Darién ecoregion, which occurs along the Pacific slope of the Andes from Panama to northwestern Ecuador and is known for its high level endemic biodiversity.

### Hydrobiosella andina sp. n.

Taxon classificationAnimaliaTrichopteraPhilopotamidae

http://zoobank.org/DB9887DE-E55B-4E9D-B4A2-1881A20FEE34

Figs 6 , 7

#### Diagnosis.

This new species is best diagnosed by its venation, elongate and relatively undifferentiated inferior appendages, simple structure of tergum X, and (especially) by the absence of preanal appendages.

#### Description.

Male. Forewing length 7.8 mm. Spur formula 2:4:4. Overall color fuscous (brownish black), antennae missing (except for scape). Head relatively small, postparietal sclerite moderate in length (shorter than diameter of eye), with 4 dark, stiff bristles subtending eye, eyes without apparent setae between facets. Palps relatively short, segment I very short, globular, segments II and IV short, subequal, II with stout apicomesal bristles, III and V moderate in length, subequal. Hind tibiae distinctly thickened and elongate. Forewing with forks I-V, hind wing with forks I-III and V (IV absent). Forewing with forks I and II slightly subsessile, crossveins *s*, *r-m*, and *m* hyaline and nearly linear, 3A looped to 2A, 2A to 1A, intersecting in proximal half of vein. Hind wing with forks I and II sessile, all 3 anal veins intersecting wing margin.

Genitalia. Sternites (through sternite V) only sparsely setose, V with conspicuous reticulate area surrounding opening of glands, which are well-developed, sternites VI-IX densely setose, with short setae; tergites subquadrate, distinctly narrower than sternites, with paired, desclerotized regions near posterior margins, each with ca. 3 prominent setae (2-5, variation), and also with sparse, minute setae surrounding the desclerotized areas, mostly confined to posterolateral margins. Segment VIII unmodified, only slightly shorter than preceding segments. Segment IX very simple in structure, subquadrate, as viewed laterally, ventral margin ca. twice length of dorsal margin, posteroventral margin slightly projecting; anterior margin nearly linear, with only slightly produced, broadly subtriangular apodemes near middle of segment; venter of segment IX with short suture from anterior margin, dorsum of segment absent (or strap-like and fused to base of tergum X). Tergum X very simple in structure, relatively elongate, narrow, and parallel-sided, apical ¼ abruptly narrowed and similarly parallel-sided, apex rounded, slightly down turned; tergum with numerous minute sensilla, most dense apically and laterally. Preanal appendages absent. Inferior appendages bi-segmented, very elongate, linear, densely setose; basal segment widest near base, narrowing apically; apical segment ca. ¾ length of basal segment, parallel-sided, apex rounded, with dense pad of short, thickened setae on mesal surface. Phallic apparatus very narrow, tubular, much shorter than inferior appendages; phallobase with usual basodorsal expansion, exposed part of endotheca distinctly expanded, apparently elongate (as judged by position of phallotremal sclerite), without spines or ornamentation. Phallotremal sclerite minute, weakly sclerotized.

Female. Unknown.

**Figure 6–7.**
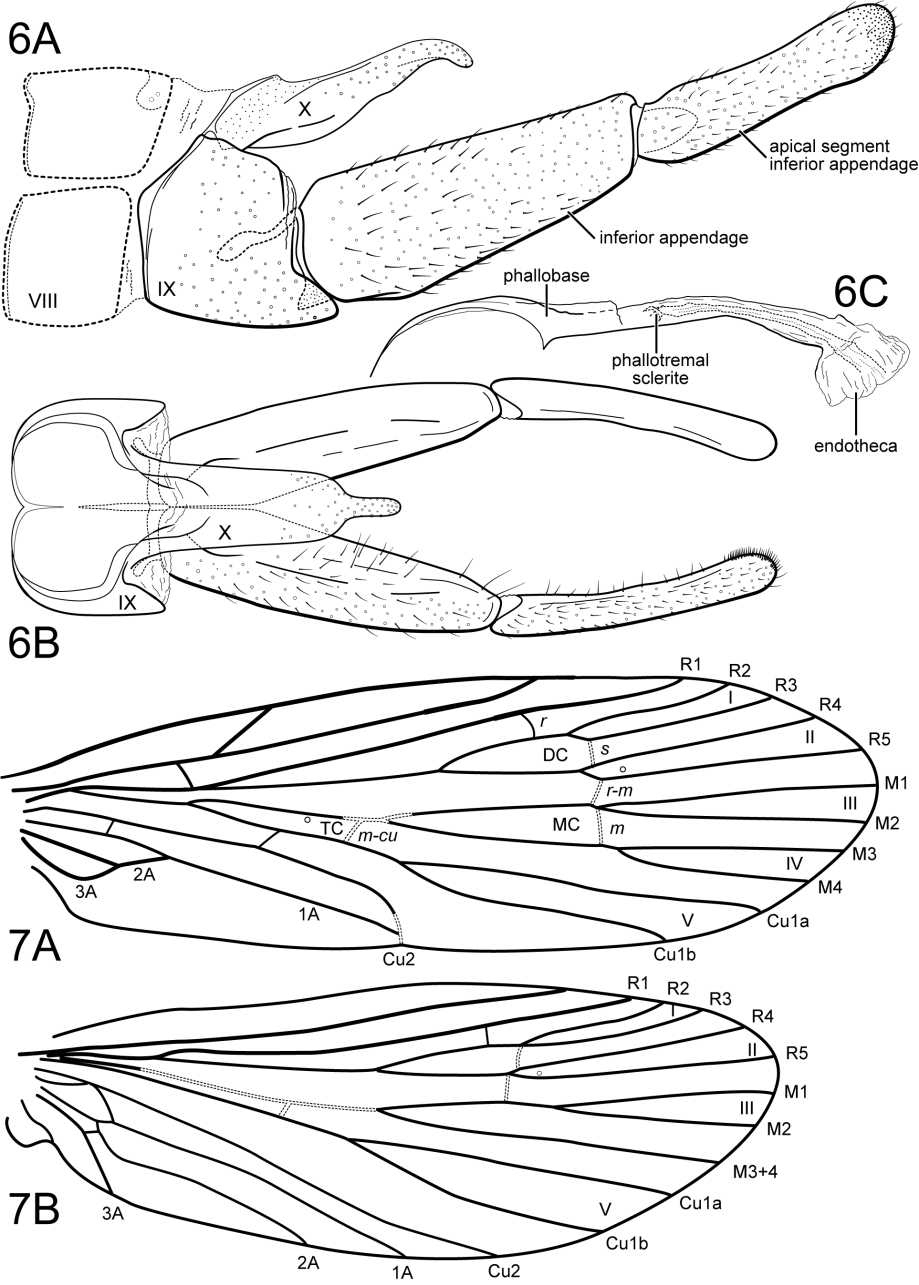
**6***Hydrobiosellaandina* sp. n. Male genitalia **A** segments VIII-X, lateral **B** segments IX-X, dorsal **C** phallus, lateral **7***Hydrobiosellaandina*, sp. n. Male wings **A** forewing **B** hind wing

#### Holotype.

**Male. ECUADOR: Tungurahua**: Río Verde, 1600 m, 26.xii.1992, VO Becker (UMSP000136163) (NMNH).

#### Etymology.

Named for the Andes Mountains, where the first specimen of this genus in the New World was discovered.

#### Remarks.

The placement of this species in *Hydrobiosella* is somewhat speculative and is based on what was considered by Ross (1956) a diagnostic feature of the genus: absence of preanal appendages (or the appendages very small or fused basally). Neboiss (1977, 1986) also used the absence or reduction of preanal appendages as a character to diagnose the Australian members of *Hydrobiosella*. In general, the genus is not very well defined and the individual species are extremely variable. We consider the placement of this new Ecuadorian species to be provisional, pending a revision of the genus. Most of the characters it possesses could be considered ancestral for Philopotaminae, including its general venation: *s*, *r-m*, and *m* crossveins of forewing hyaline and nearly linear; forks I and II of the forewing sessile or slightly subsessile; 3A of the forewing looped to 2A and 2A looped to 1A (intersecting the vein in the basal ½ of the vein, and without any cross veins); hind wing with all three anal veins reaching the wing margin. If the generic placement is correct, this represents the first record of the genus in the Americas.

### Wormaldia imbrialis sp. n.

Taxon classificationAnimaliaTrichopteraPhilopotamidae

http://zoobank.org/AB201EF5-3978-4595-88B7-A401A28B2A00

Fig. 8 , 9

#### Diagnosis.

This species is similar to *W.prolixa* Flint, *W.andrea* Muñoz-Quesada & Holzenthal, and *W.gallardoi* Muñoz-Quesada & Holzenthal, a group of Neotropical *Wormaldia* that was characterized by Muñoz-Quesada and Holzenthal (2015) as having segment IX strongly acute and projecting anterolaterally, the “head” of tergum X convexly subtriangular with its apex subtriangularly widened, and the apical segment of the inferior appendage longer than the basal segment. *Wormaldiaimbrialis*, new species, differs from this general character assessment in that segment IX is not strongly projecting anteriorly and the apical segment of the inferior appendage is shorter than the basal. Additionally, the new species differs from other species in the group in having a very long and slender phallic spine, while in the others it is very short and hooked.

#### Description.

Male. Forewing length 3.9 mm. Spur formula 2:4:4, foretibial spurs very short. Wing venation typical for *Wormaldia*: forewing with forks I-V, forks I and II slightly subsessile, *s*, *r-m*, and *m* hyaline and linear, 3A looped to 2A and 2A to 1A, intersecting vein at about midlength, crossveins absent; hind wing with forks I-III and V (fork IV absent), 2A vein absent. General color pale yellowish brown, setae of head and thorax yellowish, with several dark brown setae on postparietal sclerite; palps and antennae darker, palps brown, antennae annulated, with brown setae basally, pale setae apically. Head very short, rounded, eyes conspicuous, with short setae between facets. Postparietal sclerite very short. Both sets of palps very short.

Genitalia. Segment VIII with tergum and sternum very narrowly separated, sternum with numerous short setae, tergum with scattered short setae on posterior half; as viewed laterally, with anterior margin nearly straight, posterior margin concave (conforming with contour of anterior margin of segment IX); as viewed dorsally, with shallow, crescentic posteromesal invagination, bordered laterally by very short spine-like projections and rounded lobes lateral to these. Segment IX synscleritous, dorsal and ventral margins invaginated anteromesally, dorsal margin very short (nearly obsolete), ventral margin about as long as sternum VIII; as viewed laterally, with anterolateral margin broadly rounded, not acutely projecting, posterior margin nearly straight. Tergum X very long, narrow, apex capitate, somewhat recurved dorsally, apex with scattered small sensilla; recurved apical projection acute as viewed laterally, rounded as viewed dorsally; apex of tergum continuous with rod-like mesal ridge, extending nearly to base; basal margins of tergum with rounded (or subangular) lateral projections as viewed dorsally, each with scattered small sensilla. Preanal appendages relatively elongate, digitate, somewhat curved basally (as viewed dorsally). Inferior appendages with basal segment elongate and wide, extending beyond tergum X; apical segment ca. ⅔ length of basal segment and much narrower, tapering apically, apex rounded, mesal surface with short, thick, spine-like setae, extending for ca. apical ⅔ of segment. Phallic apparatus proportionately very large, phallobase with greatly inflated basodorsal projection, narrowing apically; internally with very elongate, narrow spine, apex of spine acuminate.

Female. Unknown.

**Figure 8.**
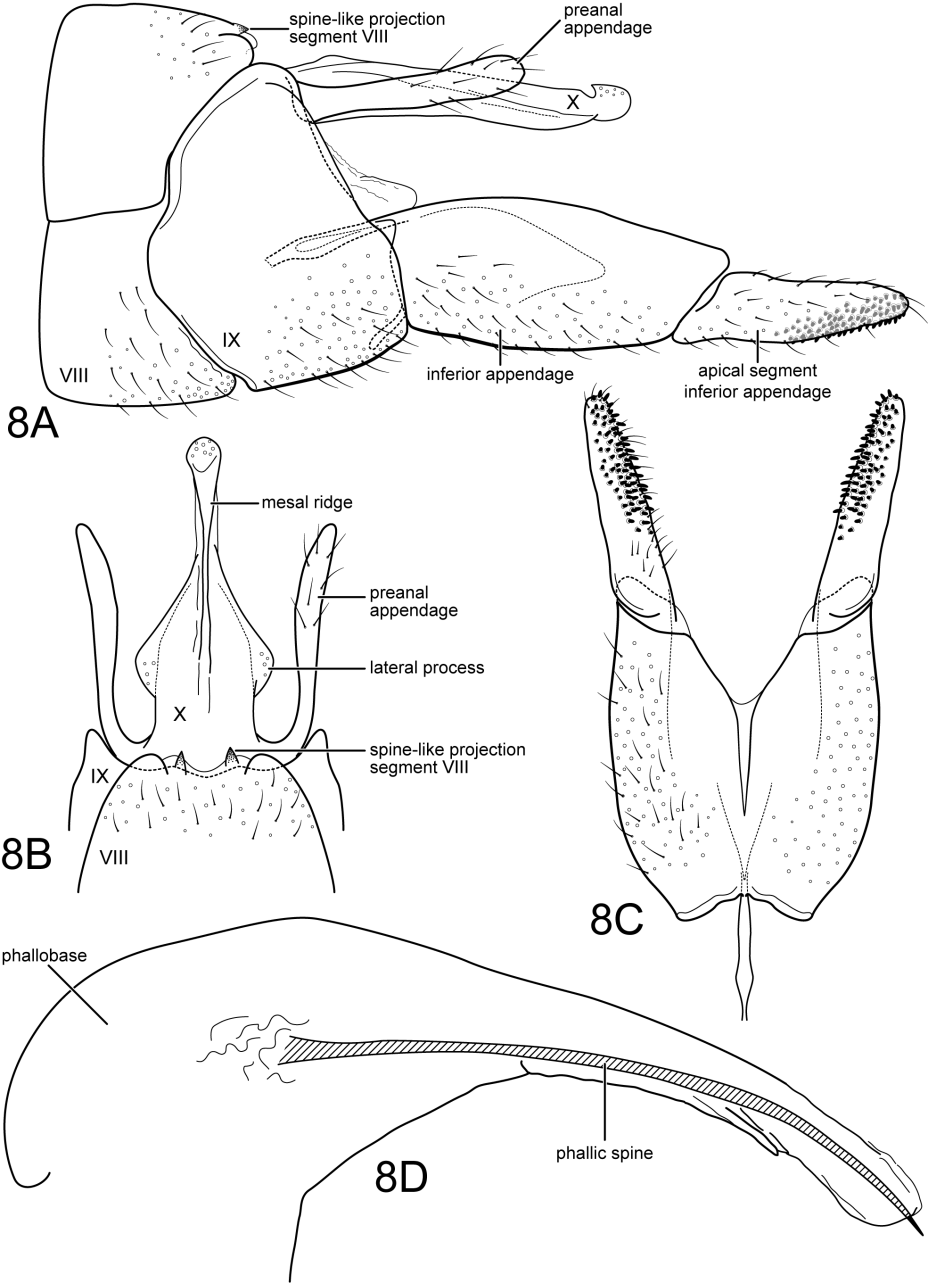
*Wormaldiaimbrialis* sp. n. Male genitalia **A** segments VIII-X, lateral **B** segments VIII-X, dorsal **C** inferior appendages, ventral **D** phallus, lateral.

**Figure 9.**
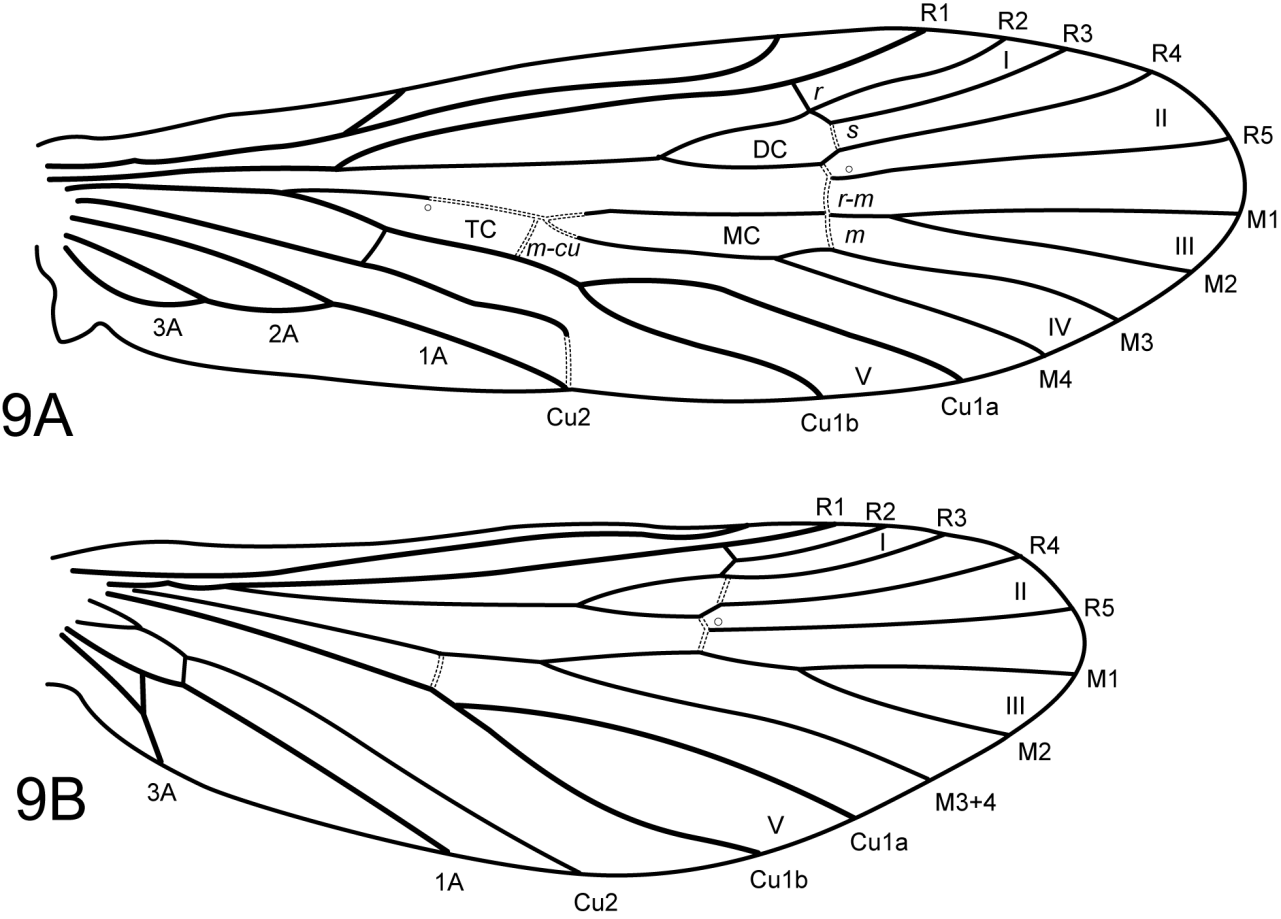
*Wormaldiaimbrialis* sp. n. Male wings **A** forewing **B** hind wing.

#### Holotype.

**Male. ECUADOR: Pichincha**: Amagusa Reserve (private), Río Mashpi Chico “alto”, 00.15487°N, 78.85316°W, 1180 m, 17.i.2015, Holzenthal, Huisman, Ríos-Touma (UMSP000146946) (UMSP).

#### Etymology.

From the Latin *imbrialis*, meaning “of rain” and referring to the night of the collection when a huge downpour occurred after the species was captured.

#### Remarks.

Despite the variation of *W.imbrialis*, from others in the *prolixa* group listed above and defined by Muñoz-Quesada and Holzenthal (2015), we note additional characters that indicate its probable inclusion in the group. All four species have similarities of tergum X and also similarities in the apical segment of the inferior appendage, including both its shape and accompanying elongate patch of short, spine-like setae. Also, each of these species has the dorsum of segment VIII with a shallow, crescentic, posteromesal invagination, bordered laterally by very short to short spine-like or thumb-like projections.

##### New record

***Chimarrhodellaperuviana*** (Ross) 1956 1956:69 [Type locality: Perú, Cusco, Paucartambo, Cosnipata Valley; INHS; ♂; in *Protarra*]. —Flint 1991:25 [♂; distribution]. —Blahnik and Holzenthal 1992:121 [♂; ♀; distribution]. —Flint 1996:385 [distribution]. —Muñoz-Quesada 2000:280 [checklist].

**New records. ECUADOR: Morona-Santiago**: tributary to Río Abanico, Hwy E46 (via Río Bamba-Macas), 2.24985°S, 78.20238°W, el. 1531 m, 12.xi.2015, Ríos-Touma, Thomson, Amigo, 2 males, 2 females (UMSP). **Sucumbios**: Reserva Municipal La Bonita, road from La Bonita to La Sofia, 0.343278°N, 77.6443721°W, el. 1416 m, 16.xi.2017, Thomson, Ríos-Touma, Amigo, 1 female (UMSP); road from La Bonita to Umpaqui, Río Seco, 0.471334°N, 77.558069°W, el. 1777 m, 17.xi.2017, Thomson, Ríos-Touma, Amigo, 1 female (UMSP).

**Distribution.** Colombia, **Ecuador**, Perú, Venezuela.

## Discussion

The description of these taxa, including a new genus and a new continental record of a formerly Australian endemic genus, indicates that there is still much to be learned and discovered of the taxonomy of the Philopotamidae of South America. Similarly, the phylogenetic and historical biogeographic relationships of the South American fauna have yet to be studied. *Hydrobiosella*, at least, shares a similar trans-Antarctic distribution pattern with Hydrobiosidae, several genera of Leptoceridae, Smicrideinae of Hydropsychidae, and *Austrotinodes*, among other groups (Cartwright 2009; Holzenthal et al. 2007; Holzenthal and Blahnik 2010). Of the other South American philopotamids, only the relationships among species of *Chimarrhodella* and *Chimarra* have been studied (Blahnik 1998; Blahnik and Holzenthal 1992; Kjer et al. 2014; Wahlberg and Johanson 2014).

## Supplementary Material

XML Treatment for Aymaradella

XML Treatment for Aymaradella boliviana

XML Treatment for Chimarrhodella choco

XML Treatment for Hydrobiosella andina

XML Treatment for Wormaldia imbrialis
